# Immunological Risk Assessment of Xenogeneic Dural Patch by Comparing with Raw Material via GTKO Mice

**DOI:** 10.1155/2022/7950834

**Published:** 2022-01-17

**Authors:** Yufeng Mu, Anliang Shao, Li Shi, Bin Du, Yongjie Zhang, Jie Luo, Liming Xu, Shuxin Qu

**Affiliations:** ^1^School of Materials Science and Engineering, Southwest Jiaotong University, Chengdu 610031, China; ^2^Institute for Medical Device Control, National Institutes for Food and Drug Control, Beijing 102629, China; ^3^Shaanxi Bioregenerative Medicine Co., Ltd., Xi'an 710100, China

## Abstract

**Objective:**

In this study, *α*-Gal epitope-deficient (GGTA1 knockout (GTKO)) mice were used to assess the immunological risks of xenogeneic dural patch by comparing with raw material.

**Methods:**

The xenogeneic dural patch (T2) was prepared from bovine pericardium (T1, raw material) through decellularization and carboxymethyl chitosan (CMCS) coating. Transmission electron microscopy (TEM) and scanning electron microscopy (SEM) were used to characterize the collagen fibers and surface microstructural changes in the T1 and T2 samples. The remnant *α*-Gal epitopes and DNA of implants were detected by standardized method. T1 and T2 were implanted subcutaneously into GTKO mice for 4 and 12 weeks, respectively, and the negative control group (Con) was only performed sham operation. The total serum antibody, anti-Gal antibody, and splenic lymphocyte subtypes were analyzed by ELISA or flow cytometry, and histological analysis of implant-tissue was performed by H&E and Masson stain.

**Results:**

TEM and Sirius red staining showed that the collagen fibers in the dural patch were closely arranged, and SEM showed that a loose three-dimensional structure was successfully constructed on the surface of the dural patch after CMCS coating. The remnant DNA in T2 was 24.64 ± 8.73 ng/mg (dry weight), and clearance of *α*-Gal epitope was up to 99.83% compared to T1. The significant increases in serum total IgM, anti-Gal IgG, and anti-Gal IgM at 4 weeks and the significant changes in anti-Gal IgG and spleen lymphocyte at 12 weeks were observed in the T1 group, but no significant change was observed in the T2 group, compared to the control group. Histological semiquantitative analysis showed severe cell and tissue responses at 4 weeks and a moderate response at 12 weeks in the T1 group, while a moderate response at 4 weeks and a slight response at 12 weeks in the T2 group.

**Conclusions:**

The results demonstrated that the xenogeneic dural patch has a lower and acceptable immunological risk compared to the raw material and control, respectively. On the other hand, it was suggested that GTKO mice are useful experimental model for immunological risk assessment of animal tissue-derived biomaterials.

## 1. Introduction

For dural injury patients, dural graft is a common surgical strategy in clinical treatment [1, 2]. However, high incidence of dural injury morbidity and shortage of autogenous graft harvest drive researcher to acquire grafts from other sources [3]. Traditional autologous and allogeneic transplantation is limited by the source shortage of the donor and potential risks of infectious diseases, which facilitate the search for suitable alternatives, such as xenograft patch [2]. Biomaterials derived from mammalian extracellular matrix (ECM) have been widely used in surgical wound repair and tissue reconstruction due to their excellent biocompatibility [4, 5]. However, the immune risk caused by animal tissue-derived biomaterials directly affected the safety and effectiveness of these materials and limited their applications [4, 6, 7]. Immunological responses between the antigen on xenografts and the antibody in humans may lead to immune-mediated tissue degeneration and dystrophic calcification [8]. With the development of antigen removal technology, xenogeneic antigens can be removed effectively. However, it is difficult to completely remove all of xenogeneic antigens [8, 9]. Residual xenoantigens are still important factors that cause chronic immune rejection and affect tissue regeneration and reconstruction [10–12].

Many studies showed that the main target antigen for hyperacute rejection of xenotransplantation is *α*-Gal epitope [13–15]. The *α*-Gal epitope is a galactose protein or glycolipid, which is known to be widely expressed in mammals except humans, old world monkey, and ape, and it is mainly regulated by glycoprotein alpha-galactosyltransferase 1 (GGTA1) gene. Due to the fact that *α*-Gal epitope exists in most wild-type experimental animals, it is obviously unreasonable to use wild-type experimental animal to evaluate the *α*-Gal epitope-related immunological risks of animal tissue-derived biomaterials [16].

Tearle et al. [17] developed a GGTA1 knockout (GTKO) mouse model used for investigation of the rejection in xenotransplantation. Tanemura et al. [11] showed that rabbit red blood cells (RRBC, *α*-Gal epitope contained) can induce GTKO mice to produce high titers of anti-Gal IgG and IgM, indicating that GTKO mice caused a sensitive immunogenic response to the stimulation of *α*-Gal epitope-positive material. Our group also developed GTKO mice which have been used to evaluate the immunological risks of various animal tissue-sourced decellularized materials [18, 19].

In this study, the immune risk of xenogeneic dural patch was evaluated by subcutaneous implantation experiment using GTKO mice for 4-week and 12-week periods, by comparing with raw material (bovine pericardium).

## 2. Materials and Methods

### 2.1. Material Preparation

The xenogeneic dural patch material is a composite of decellularized bovine pericardium tissue and carboxymethyl chitosan (CMCS) coating, which was provided by Shaanxi Bioregenerative Medicine Co., Ltd. The main manufacture processes of xenogeneic dural patch were as follows. Briefly, in order to decellularize and remove potential antigens, the pericardium tissue was repeatedly frozen-thawed and crushed to remove the cells, and then, a cross-linking agent is used to fix the extracellular matrix. And then, CMCS was coated to the one-side surface of the membrane tissue with spin coating technology. For the CMCS coating, 0.2% CMCS and 2% hydroxypropyl methyl cellulose were mixed into a homogeneous solution, and 0.1% (*w*/*v*) EDC solution was added to form a cross-linking solution under magnetic stirring. After 1 hour of cross-linking, the mixed solution was spread on the decellularized bovine pericardium at room temperature. Finally, the xenogeneic dural patches (T2) were obtained after deep-low temperature freeze-drying and ^60^Co sterilization and the detailed processes shown in the patent of Shaanxi Bio-Regen Med (CN 102727935 B, CN 106310373 A).

The bovine pericardia (T1, raw material) were mainly prepared by the process of physical cutting, freeze-drying, packaging, and sterilization and were used as positive control in this study.

### 2.2. Material Characterization

#### 2.2.1. Morphological Characterization

For TEM observation, the specimens were fixed with 2.5% of glutaraldehyde, then stained with 1% OsO4, and then dehydrated with graded ethanol, and embedded in EPON812/DDSA/MNA/DMP-30. Thin sections were stained with uranyl acetate and lead citrate and observed with a TEM (H-7650 Tokyo, Japan) at 80 kV.

For SEM observation, a thin layer of gold (approx. 100 Å) was coated on the T1 and T2 specimens to increase conductivity by a sputter coater (K575X, Emitech, UK) and characterized by a scanning electron microscope (SEM, Carl Zeiss AG, Germany) under an accelerating voltage of 5 or 20 kV.

#### 2.2.2. Sirius Red Staining

The specimens were routinely dehydrated and embedded in paraffin, 6 *μ*m sectioned and deparaffinized, and stained with Weigert iron hematoxylin staining solution for 10-20 min. Differentiate in acidic differentiation solution for a few seconds, wash with water for 5-10 minutes, and wash once with distilled water. After staining with Sirius red staining solution for 1 h, rinse it with water to remove the staining solution on the surface of the section. And then, the sections were dehydrated and transparent, sealed with neutral gum, and observed under a microscope.

#### 2.2.3. Remnant DNA Determination

The remnant DNA of xenogeneic dural patch was determined according to the Chinese industry standard “tissue engineered medical products—part 25: determination of DNA residues in animal tissue derived biomaterials: fluorescence staining method” (YY/T 0606.25). Before detection, the sample was dried by vacuum until it reaches constant weight which will be used for the sample dry weight in calculating remnant DNA content (ng/mg dry weight). Briefly, making DNA release from ECM materials by proteinase k digestion or/and combined with tissue homogenization, the released DNAs were subsequently purified by using nucleic acid extraction kit (4400793, 4400795, 4400675, ABI) and DNA determination using fluorescence assay with PicoGreen dsDNA reagent (P7589, Invitrogen).

#### 2.2.4. *α*-Gal Epitope Determination

The remnant *α*-Gal epitope of xenogeneic dural patch and bovine pericardium was determined referring to the Chinese industry standard “tissue engineering medical device products—remnant a-Gal epitope determination in scaffold materials utilizing animal tissues and their derivatives” via a standardized ELISA inhibition assay [20–22] using a commercial *α*-Gal epitope detection kit (Meitan 70101, Beijing Sanyao Science & Technology Development Co., Beijing, China). Briefly, a calibration curve was produced using the Gal-BSA/Gal-free matrix (*α*-Gal epitope-negative biomaterial reference material, 380003-201701, provided by National Institutes for Food and Drug Control (NIFDC), China) as a *α*-Gal epitope reference material. An *α*-Gal epitope-positive biomaterial reference material (380003-201701, provided by NIFDC, China) was used as a positive control to monitor the sensitivity of the test system, and an *α*-Gal epitope-negative biomaterial reference material was used as a negative control to monitor the specificity of the test system. Before detection, the sample was dried by vacuum until it reaches constant weight which will be used for the sample dry weight in calculating remnant *α*-Gal epitope content (epitopes/dry weight). For all samples, the lysates were prepared by homogenizing them in certain amount of lysis buffer (e.g., 5-10 mg/mL) containing 1% protease inhibitor PMSF using a homogenizer (Benchmark D1000-E), incubating at room temperature for 3 h, and making sure that there were no obvious solid matter and the *α*-Gal epitope was completely released. All samples centrifuged at 4000 rpm for 10 min, 200 *μ*L volume supernatant per sample was obtained. All supernatant (200 *μ*L) of test samples was incubated with the primary monoclonal antibody M86 (200 *μ*L) for 2 h at 37°C with gentle shaking and then left at 4°C overnight. Before use, the reaction mixtures were centrifuged at 14,000 *g* for 30 min at 4°C, and 100 *μ*L/well of the supernatant containing residual M86 antibody was loaded into a Gal-BSA precoated 96-well plate and incubated for 1 h with gentle shaking at 37°C. After washing, 100 *μ*L of secondary horseradish peroxidase- (HRP-) conjugated antibody was loaded and incubated for 30 min. Finally, following washing, 100 *μ*L of HRP color development solution was added to each well for 15 min at room temperature (keep in dark place). The reaction was stopped by 50 *μ*L of 10% H_2_SO_4_. The absorbance was measured by a microplate reader (Spectramax M5, Molecular Devices, CA) at 450 nm.

### 2.3. Preparation of GTKO Mice

The 5-week-old GGTA1 knockout (GTKO) mice were provided from the Institute for Laboratory Animal Resources/NIFDC, China [18]. All animals were maintained in a specific pathogen-free facility under the following conditions: 23 ± 1°C, relative humidity of 30%-70%, and 12 h light/12 h dark cycle. Animals were housed and handled in accordance with the guidelines set by the Association for the Assessment and Accreditation of Laboratory Animal Care. The study was approved by the NIFDC Institutional Animal Care and Use Committee.

### 2.4. Implantation Experiment and Sampling

All mice were preimmunized twice with RRBC membrane (1 × 10^8^ RRBCs) via intraperitoneal injection at 6 weeks and 8 weeks. One week after the last treatment, total 60 GTKO mice including 30 female and 30 male mice were divided into 6 groups (10 per group, 5 female and 5 male) used for three experimental groups: bovine pericardium (T1, positive control), xenogeneic dural patch (T2), and negative control (Con) for 4- and 12-week implantation periods, respectively. Before implantation, all animals were anesthetized with 1.5% pentobarbital sodium (50 mg/kg), and implants (1 cm × 3 cm) were implanted subcutaneously in the dorsal area of the GTKO mice, while the negative control group was only performed sham operation. The serum, spleen, and local implants containing tissue from all groups were collected at 4 weeks and 12 weeks postimplantation for immunological assessments and pathological analysis.

### 2.5. Detection of Total Serum IgG and IgM

Total serum IgG and IgM level of mice after implantation was assessed via ELISA according to the manufacturer's protocol. Briefly, total serum IgG was detected using a mouse IgG (total) ELISA Kit (EMC116, Neo-bioscience, Beijing, China) and serum samples were diluted at 1 : 4 × 10^6^. Total serum IgM was detected using a mouse IgM ELISA Kit (EMC129, Neo-bioscience, Beijing, China), and serum samples were diluted at 1 : 6 × 10^4^. The optical density (OD) value was read at 450 nm using a microplate reader (Spectramax M5, Molecular Devices, CA, USA).

### 2.6. Detection of Serum Anti-Gal IgG and IgM

The detection of anti-Gal IgG and IgM in mouse serum was carried out via ELISA according to the instructions of the manufacturer's protocol. Briefly, anti-Gal IgG was detected using a mouse anti-Gal IgG ELISA Kit (ZCT1218, Customized, Wantai Biopharm, Beijing, China) and serum samples were diluted at 1 : 20. Anti-Gal IgM was detected using a mouse anti-Gal IgM ELISA Kit (ZCT1217, Customized, Wantai Biopharm, Beijing, China), and serum samples were diluted at 1 : 200. The optical density (OD) value was read at 450 nm using a microplate reader.

### 2.7. Splenic Lymphocyte Subtype Analysis

The spleens were carefully dissected after mice were sacrificed at 4 weeks and 12 weeks. Spleen tissues were minced, homogenized, and filtered through a 70 *μ*m cell strainer. The cell suspensions were centrifuged at 300 *g* for 5 min. Centrifugal precipitates were resuspended in 1640 cell culture medium and recentrifuged under the same conditions to obtain splenic mononuclear cells. Mononuclear cells were resuspended in 1640 cell medium at a concentration of 1 × 10^6^ cells/mL. Each of the labeled antibodies (FITC, anti-mouse CD3/PE, anti-mouse CD8a/PE, anti-mouse CD69/PerCP, anti-mouse CD45/APC, anti-mouse CD19/APC, and anti-mouse CD49b) was added to each flow tube, respectively. A splenic cell suspension of each sample (100 *μ*L) with a reasonable concentration was added to the flow tube. After thorough mixing, the samples were incubated at room temperature for 20 min keeping in the dark place. Next, the mixture was washed with 2 mL of PBS and centrifuged at 300 *g* for 5 min, and the supernatant was discarded. After resuspension of the pellet in 0.5 mL of PBS, cells were detected via flow cytometry (Canto II, BD, New York, USA).

### 2.8. Histological Analysis of Implants and Tissues

Histological analysis of implants and local tissues from all three groups was performed using both hematoxylin and eosin (H&E) staining and Masson staining. Briefly, implant-tissues were dissected and fixed with 10% buffered formalin solution for 48 h. Samples were subsequently dehydrated using alcohol and xylene and embedded in paraffin wax. All specimens were sliced into 5 *μ*m thick sections for H&E and Masson staining, respectively. Stained slides were observed under a light microscope.

### 2.9. Statistical Analysis

Numerical data are presented as means ± SD unless otherwise indicated. Data were analyzed via one-way analysis of variance (ANOVA, Tukey's post hoc analysis) using statistical package SPSS 20.0 (SPSS Inc., Chicago, IL, USA). Statistical significance was set at ^∗^*p* < 0.05 and ^∗∗^*p* < 0.01 versus the indicated group.

## 3. Results

### 3.1. Morphological Characterization

**Figure 1:**
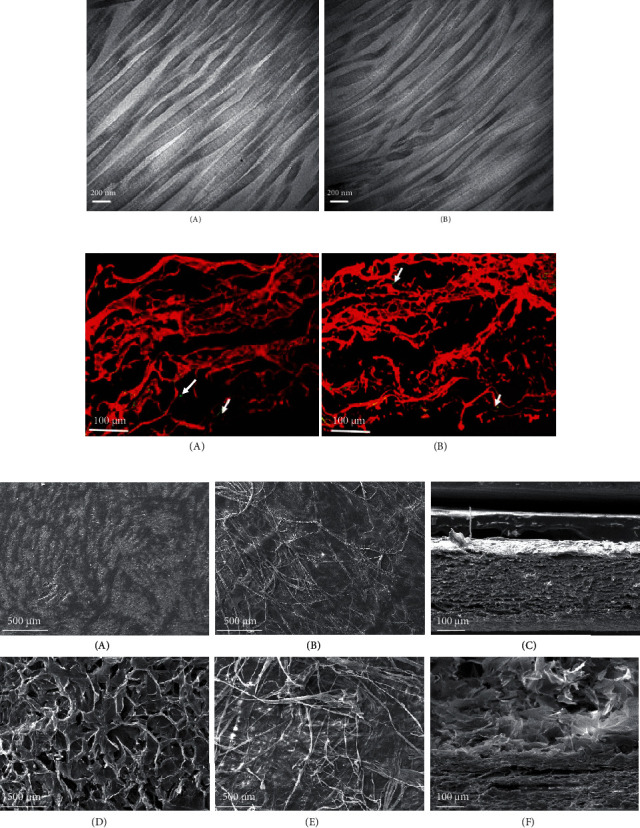
(a) The TEM graphs of dural patch: (A) bovine pericardium before decellularization; (B) bovine pericardium after decellularization. The image was taken at ×50,000 magnification. Scale bar: 200 nm. (b) The Sirius red staining image of dural patch: (A) bovine pericardium before decellularization; (B) bovine pericardium after decellularization. The image was taken at ×400 magnification. Scale bar: 100 *μ*m. The arrows show green fine fiber, representing type III collagen. (c) SEM micrographs of dural patch. Images (A), (B), (D), and (E) were taken at ×100 magnification, and images (C) and (F) were taken at ×300 magnification.

The TEM graphs showed the morphological characteristics of the bovine pericardium before and after experimental treatments, as shown in Figure 1(a). Periodic transverse/lamellar structures are clearly seen along the long axis of collagen fibers; decellular treatment did not change the structure of collagen fibers.

As showed in Figure 1(b), Sirius red staining before and after decellularization showed that decellular treatment did not change the collagen structure in the dural patch which composed with mainly type I collagen (presented as yellow or red fiber) and a small amount of type III collagen (presented as green fine fibers).

The SEM images showed the different morphological characteristics of the xenogeneic dural patch before and after CMCS coating, as shown in Figure 1(c).

Before the CMCS coating, the tissue structure of the bovine pericardium clearly presented a serosal surface (A) and a dense collagen fiber surface (B). The cross-sectional view of the bovine pericardium showed that the pericardial tissue presents a relatively regular slice structure (C). After being coated with CMCS, the bovine pericardium serosal surface presented an obvious three-dimensional porous structure which presented as the loose surface of the patch (D). The arrangement of collagen fibers in the pericardium did not change significantly before (B) and after (E) CMCS coating. The cross-sectional view (F) of the patch also showed the obvious structural changes between the coating and the substrate.

### 3.2. Remnant DNA and *α*-Gal Epitope Detection

The remnant DNA of the decellularized bovine pericardium which was used for preparation of xenogeneic dural patch was 24.64 ± 8.73 ng/mg (dry weight), and the *α*-Gal epitope content in bovine pericardium (T1) was (5.04 ± 0.78) × 10^15^/mg (epitopes/dry weight) and in xenogeneic dural patch (T2) was (8.35 ± 0.27) × 10^12^/mg (epitopes/dry weight), as showed in Table 1. The clearance rate of *α*-Gal epitope in T2 was up to 99.83% compared to T1, which demonstrated that the treatment of decellularization and antigen removal processes efficiently removed the *α*-Gal epitope from the bovine pericardium.

### 3.3. Total Serum IgG and IgM

**Figure 2:**
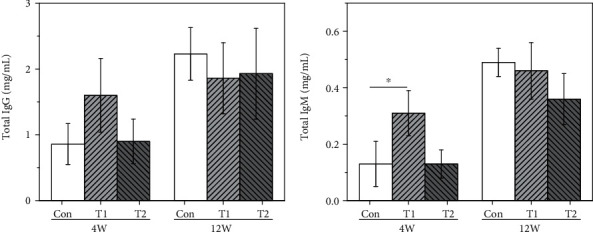
Total serum IgG and IgM levels in the three groups at 4 and 12 weeks postimplantation (^∗^*p* < 0.05): (a) total IgG and (b) total IgM.

After implantation of 4 and 12 weeks, the levels of total serum IgG and IgM from the three groups are shown in Figure 2.

The total IgM in the T1 group were significantly higher than those in the control group (*p* < 0.05) at 4 weeks postimplantation. But there were no statistical differences in total serum IgG and IgM found in the T2 group compared to the control group at 4 and 12 weeks postimplantation.

### 3.4. Anti-Gal IgG and IgM

**Figure 3:**
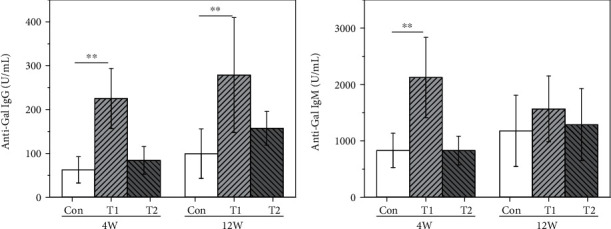
The levels of anti-Gal antibodies in the three groups at 4 and 12 weeks postimplantation (^∗∗^*p* < 0.01): (a) anti-Gal IgG and (b) anti-Gal IgM.

The levels of anti-Gal IgG and IgM are shown in Figure 3. At 4 weeks postimplantation, the serum anti-Gal IgG and IgM in the T1 group were significantly increased (about 3.6 times higher for IgG, 2.6 times higher for IgM) compared with those in the control group. At 12 weeks postimplantation, the serum anti-Gal IgG of the T1 group was still 2.8 times higher than that in the control group. In contrast, no statistical differences can be found in the anti-Gal IgG and IgM levels in the T2 group at any implantation period compared to the control group. These results demonstrated that T2 did not cause systematic anti-Gal responses at any implantation period.

### 3.5. Spleen Lymphocyte Subtype Analysis

#### 3.5.1. T Lymphocytes

**Figure 4:**
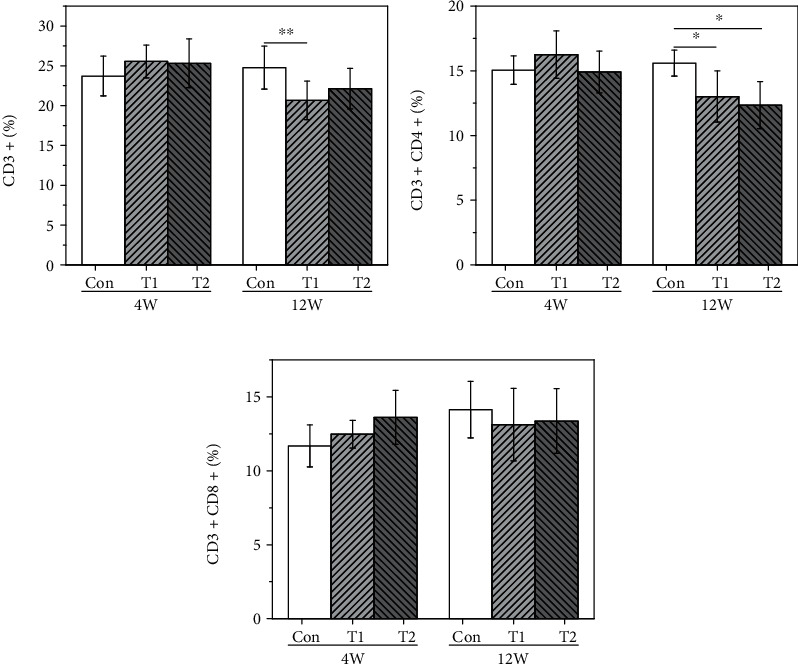
The proportion of T lymphocyte subtype in the three groups at 4 and 12 weeks postimplantation (^∗^*p* < 0.05; ^∗∗^*p* < 0.01): (a) CD3^+^, (b) CD3^+^CD4^+^, and (c) CD3^+^CD8^+^.

T lymphocyte subtypes were analyzed by flow cytometry, and the results are shown in Figure 4. At 4 weeks postimplantation, T lymphocyte subtypes in the T1 and T2 groups showed no statistical difference compared to the control group. At 12 weeks postimplantation, the proportion of CD3^+^ cells and CD3^+^CD4^+^ cells in the T1 group was significantly lower than that in the control group. At the same time, CD3^+^CD4^+^ in the T2 group was significantly lower than that in the control group.

#### 3.5.2. B Lymphocytes

**Figure 5:**
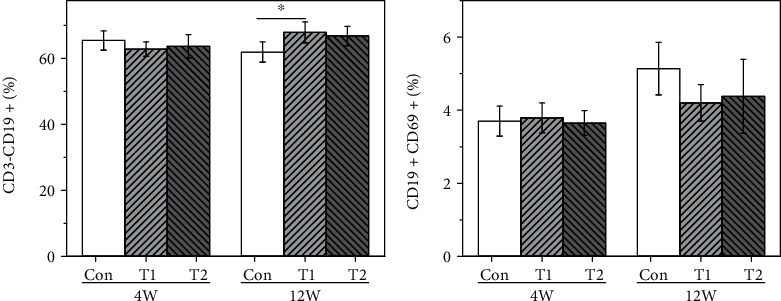
The proportion of B lymphocyte subtype in the three groups at 4 and 12 weeks postimplantation (^∗^*p* < 0.05): (a) CD3^+^CD19^+^ and (b) CD19^+^CD69^+^.

B lymphocyte subtypes were analyzed by flow cytometry, and the results are shown in Figure 5. At 4 weeks postimplantation, B lymphocyte subtypes in the T1 and T2 groups showed no statistical difference compared to those in the control group. At 12 weeks postimplantation, the proportion of CD3^−^CD19^+^ subtype in the T1 group was significantly higher than that in the control group, but no differences were found in the T2 group compared to the control.

#### 3.5.3. NK Lymphocytes

**Figure 6:**
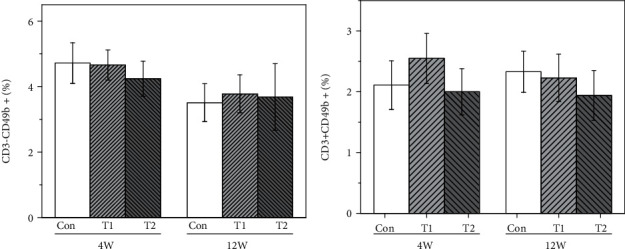
The proportion of NK lymphocyte subtype in the three groups at 4 and 12 weeks postimplantation: (a) CD3^−^CD49b^+^ and (b) CD3^+^CD49b^+^.

NK lymphocyte subtypes were analyzed by flow cytometry and the results are shown in Figure 6, and there were no significant differences found in both T1 and T2 groups compared to the control at 4 and 12 weeks postimplantation.

### 3.6. Pathological Analysis

**Figure 7:**
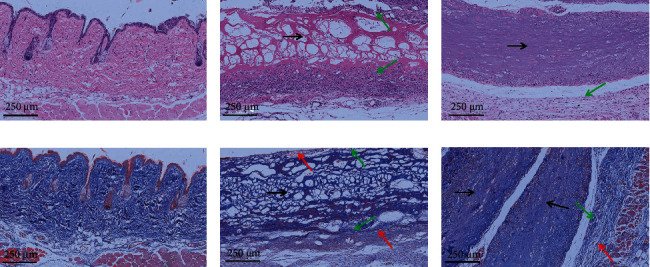
H&E and Masson staining images of implant-tissues at 4 weeks: (a–c) H&E-stained images of the control, T1, and T2 groups, respectively; (d–f) Masson-stained images of the control, T1, and T2 groups, respectively. The bar is 250 *μ*m. The black arrows indicate the implant, the green arrows indicate the fibrous capsule, and the red arrows indicate infiltrating inflammatory cells.

**Figure 8:**
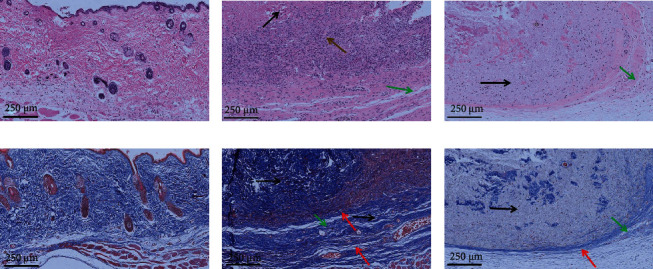
H&E- and Masson-stained images of implant-tissues at 12 weeks: (a–c) H&E-stained images of the control, T1, and T2 groups, respectively; (d–f) Masson-stained images of the control, T1, and T2 groups, respectively. The bar is 250 *μ*m. The black arrows indicate the implant, the green arrows indicate the fibrous capsule, and the red arrows indicate infiltrated inflammatory cells.

The images of H&E and Masson staining of the implant-tissues are shown in Figures 7 and 8. At 4 weeks postimplantation, the implants in the T1 and T2 groups remained intact, and no obvious degradation was observed. But there was no CMCS coating observed in CMCS-coated dural patch. At 12 weeks postimplantation, still no significant degradation was observed. Another degradation experiment showed that degradation of dural patch was very slow; about 50% degradation was observed after 6 months posttransplantation (unpublished data).

Semiquantitative evaluation of pathological sections was performed to observe the inflammation and tissue responses at the implantation site of GTKO mice by referring to ISO10993-6:2016 [23]. As showed in Tables 2 and 3, at 4 weeks postimplantation, macrophages, neutrophil infiltration and lymphocyte infiltration, remarkable angiogenesis, and fibrous capsule formed around the implant were observed, and the comprehensive semiquantitative score was 16.3, indicating that it was a severe cell and tissue response in the T1 group. The T2 group also showed inflammation infiltration, angiogenesis, and fibrous encapsulation, but the semiquantitative score was 9.7, indicating that it was a moderate cell and tissue response. At 12 weeks postimplantation, the semiquantitative score in the T1 group was 10.6, indicating that it was still a moderate cell and tissue response, but the semiquantitative score was 8.7 in the T1 group indicating that it was a slight cell and tissue response.

## 4. Discussions

The *α*-Gal epitope is known to exist in all mammals except humans, old world monkey, and ape, and it is one of target antigens that could cause hyperacute immune rejection in the human body [15]. In this study, the xenogeneic dural patch was sourced from bovine pericardium. After decellular and antigen removal processes, the clearance rate of *α*-Gal epitope was achieved up to 99.83%, and the remnant DNA was 24.64 ± 8.73 ng/mg (dry weight) which was less than a generally considered criteria 50 ng/mg. The decrease of *α*-Gal epitope will help to reduce the immune rejection response of animal tissue-derived biomaterials on human host [11, 24], while the ECM structure and main components such as collagens in xenogeneic dural patch were maintained very well as showed in the SEM images, TEM images, and Sirius red staining image, and the structural integrity will provide a good mechanical property for clinical application. In order to facilitate the repair of the dural defect, the CMCS coating was applied to one side of the decellularized bovine pericardium. In clinical use, CMCS can form a gel after being in contact with blood or tissue fluid, which can be attached to the wound surface to seal it and prevent cerebrospinal fluid leakage and tissue adhesion [25–27].

Due to the fact that *α*-Gal epitope exists in most wild-type experimental animals, it is obviously unreasonable to use wild-type experimental animal to evaluate the *α*-Gal epitope-related immunological risks of animal tissue-derived biomaterials [15, 28]. To evaluate the risks of systemic immune rejection responses [29] of xenogeneic dural patch objectively, *in vivo* implantation experiment via GTKO mice was performed in this study, and to compare the effectiveness of decellular processes, raw materials were used as *α*-Gal epitope-positive control. The raw material (T1 group, bovine pericardium) showed higher titers in total antibody and anti-Gal antibody at early implantation (4 weeks), and the anti-Gal IgG even continued to rise at 12 weeks. Meanwhile, lymphocytes were also thought to be involved in host foreign body responses mediated by biological materials [30–32]; in this research, B lymphocyte subtype CD3^−^CD19^+^ and NK lymphocyte subtype CD3^+^CD49b of the spleen were significantly higher than those in the control. In the semiquantitative scoring system for inflammation and tissue response evaluation, the T1 group also showed a severe reaction at 4 weeks and a moderate reaction at 12 weeks. These results suggested that the GTKO mice are sensitive to animal tissue-induced immunological responses, especially *α*-Gal epitope-related immunological responses.

Comparing to raw material, the xenogeneic dural patch (T2 group) did not cause significant increase in total antibodies, specific anti-Gal antibodies, and splenic lymphocyte subtypes, suggesting that the decellular and antigen removal processes of bovine pericardium for xenogeneic dural patch preparation effectively decreased immunological response risks. These results are consistent with the result of the remnant DNA less than 50 ng/mg, and the clearance rate of *α*-Gal epitope was up to 99.83% in xenogeneic dural patch. ECM has many unique advantages, such as good biocompatibility, excellent biomechanical properties, and rich in cell growth factors [33–35]. However, the immune response caused by ECM, such as DNA, *α*-Gal epitope, or residual glutaraldehyde [36, 37], should not be ignored. Currently, it is not clear whether there is a threshold of Gal epitope stimulation in the immune response of xenotransplantation induced by *α*-Gal epitope. Galili et al. [15, 33] pointed out that a 95% reduction in the expression of *α*-Gal epitope in pig cells may still cause the host immune response.

At 4 weeks postimplantation, although the T2 group also showed inflammation and tissue response, the semiquantitative score result was a moderate response at 4 weeks and a slight response at 12 weeks. The CMCS coating was not likely to be a reason for causing early (4 weeks) acute inflammatory response; this also was evidenced by Cy5 dye-leveled CMCS metabolism study recorded by fluorescence intensity via in vivo imaging; in that, CMCS degradation and metabolism reached the peak in 7-14 days accumulating in the liver and 72 h-7 days accumulating in the kidney, and fluorescence intensity significantly decreased at 4 weeks (unpublished data). As generally understood, in addition to the residual xenoantigen, any residual chemical reagent (used for acellular, inactivated virus, etc.) may cause varying degrees of inflammatory reaction to the host, and all the foreign grafts implanted into the host may cause foreign body response (FBR) [38–40]. The FBR at 12 weeks was reduced than that at 4 weeks and mainly showed neovascularization, fibrosis, and slight macrophage, which are also considered as positive tissue responses indicating tissue regeneration process [35, 41, 42]. The above results suggested that after decellularization treatment and CMCS coating, the residual immunogenicity risk of the xenogeneic dural patch seems to be acceptable. In order to further verify the clinical application prospects of xenogeneic dural patch, it is necessary to carry out in situ repair experiments of dural mater defects based on larger animals.

In conclusion, this study investigated the immunological risk of decellularized bovine pericardium-sourced dural patch via GTKO mice. The results indicated that the xenogeneic dural patch did not cause significant immunological responses to GTKO mice, suggesting that decellular and antigen removal processes for xenogeneic dural patch preparation effectively decreased immune response risk. On the other hand, it was suggested that the GTKO mice are good experimental animal model which could be used for undesirable immunological response risk assessment of animal tissue-derived biomaterials.

## Figures and Tables

**Table 1:** The DNA and *α*-Gal epitope content.

Sample	Remnant DNA content (ng/mg dry weight)	*α*-Gal epitope content (epitopes/mg dry weight)
T1	/	(5.04 ± 0.78) × 10^15^
T2	24.64 ± 8.73	(8.35 ± 0.27) × 10^12^

**Table 2:** Histological evaluation: cell type and tissue response scoring scheme (mean ± SE, *n* = 10).

Groups	At 4 weeks			At 12 weeks		
	Con	T1	T2	Con	T1	T2
Polymorphonuclear cells	0	2.1 ± 0.3	0.6 ± 0.3	0	1.0 ± 0.4	0
Lymphocytes	1.0 ± 0.0	1.2 ± 0.1	1.1 ± 0.1	1.0 ± 0.0	1.0 ± 0.0	1.0 ± 0.0
Plasma cells	0	0	0	0	0	0
Macrophages	0	2.9 ± 0.1	2.3 ± 0.2	0	1.8 ± 0.2	1.5 ± 0.3
Giant cells	0	0	0.2 ± 0.2	0	0	0
Necrosis	0	0	0.2 ± 0.2	0	0	0
Neovascularization	0	1.7 ± 0.2	1.0 ± 0.0	0	1.1 ± 0.1	1.0 ± 0.0
Fibrosis	0	2.2 ± 0.1	2.0 ± 0.0	0	2.0 ± 0.2	1.1 ± 0.1
Fatty infiltrate	0	0	0	0	0	0.8 ± 0.3

**Table 3:** Summarized semiquantitative scoring scheme of histological evaluation.

Group		Inflammation (×2)	Neovascularization	Average^a^	Number of implants examined^b^
At 4 w	Con	20	0	2	20
	T1	124	39	16.3	20
	T2	70	27	9.7	20
At 12 w	Con	20	0	2	20
	T1	68	28	10.6	20
	T2	58	29	8.7	20

^a^Used to determine reactivity ranking shown below as the conclusion. A negative difference is recorded as zero. ^b^Histological evaluation score represents the averaged score for that animal across the number of implants examined.

## Data Availability

The data used to support the findings of this study are included within the article.
